# RNAseq Reveals the Contribution of Interferon Stimulated Genes to the Increased Host Defense and Decreased PPR Viral Replication in Cattle

**DOI:** 10.3390/v12040463

**Published:** 2020-04-20

**Authors:** Krishnaswamy Gopalan Tirumurugaan, Rahul Mohanchandra Pawar, Gopal Dhinakar Raj, Arthanari Thangavelu, John A. Hammond, Satya Parida

**Affiliations:** 1 Department of Animal Biotechnology, Madras Veterinary College, Tamil Nadu Veterinary and Animal Sciences University, Chennai 600007, India; tirumurugaan.k.g@tanuvas.ac.in (K.G.T.); rahul.abt@gmail.com (R.M.P.); 2 Centre for Animal Health Studies, Tamil Nadu Veterinary and Animal Sciences University, Chennai 600051, India; 3 Department of Veterinary Microbiology, Madras Veterinary College, Tamil Nadu Veterinary and Animal Sciences University, Chennai 600007, India; thangavelu.a@tanuvas.ac.in; 4 The Pirbright Institute, Ash Road, Pirbright, Surrey GU24 0NF, UK; john.hammond@pirbright.ac.uk

**Keywords:** RNAseq, differential PPR disease resistance, differentially expressed, interferon stimulated genes (ISGs), PDE12

## Abstract

*Peste des petits ruminants virus* (PPRV) is known to replicate in a wide variety of ruminants causing very species-specific clinical symptoms. Small ruminants (goats and sheep) are susceptible to disease while domesticated cattle and buffalo are dead-end hosts and do not display clinical symptoms. Understanding the host factors that influence differential pathogenesis and disease susceptibility could help the development of better diagnostics and control measures. To study this, we generated transcriptome data from goat and cattle peripheral blood mononuclear cells (PBMC) experimentally infected with PPRV in-vitro. After identifying differentially expressed genes, we further analyzed these immune related pathway genes using the Search Tool for the Retrieval of Interacting Genes/Proteins (STRING) and selected candidate genes were validated using in-vitro experiments. Upon PPRV infection, we identified 12 and 22 immune related genes that were differentially expressed in goat and cattle respectively. In both species, this included the interferon stimulated genes (ISGs) IFI44, IFI6, IFIT1, IFIT2, IFIT3, ISG15, Mx1, Mx2, OAS1X, RSAD2, IRF7, DDX58 and DHX58 that were transcribed significantly higher in cattle. PPRV replication in goat PBMCs significantly increased the expression of phosphodiesterase 12 (PDE12), a 2′,5′-oligoadenylate degrading enzyme that contributes to the reduced modulation of interferon-regulated gene targets. Finally, a model is proposed for the differential susceptibility between large and small ruminants based on the expression levels of type-I interferons, ISGs and effector molecules.

## 1. Introduction

*Peste des petits ruminant’s virus* (PPRV), a morbillivirus in the family Paramyxoviridae, causes an acute, extremely contagious disease. Ovine rinderpest or goat plaque is characterized by high fever, ocular and nasal discharges, pneumonia, necrotic and inflammation and ulcerative lesion of the mucosa in the gastrointestinal tract [1]. PPRV was first reported in India in 1989 and subsequently spread all over the country [2,3,4,5]. In India, the disease is mainly controlled through the use of a Vero cell-attenuated Sungri 96 vaccine which elicits a protective antibody response forup to 78 months [6]. PPRV infection is usually confined to populations of small ruminants with particular breeds of goats being reported as more susceptible than others [7] and with more severe pathology compared to sheep [8,9]. Differential disease resistance to PPRV has been reported both at the species and breed levels; the Guinean breeds (West African dwarf (WAD), Iogoon, Kindi and Djallonke) are known to be highly susceptible [7].

Although Cattle can become infected with PPRV, unlike the closely related rinderpest virus (RPV), they do not show clinical signs and are not susceptible to disease [7,10]. However, virus replication and sero-conversion does occur in large ruminants [11]. Interestingly, a clinical case of PPRV infection was reported following experimental inoculation of calves [12] and another report describes that PPRV was isolated from an RPV-like outbreak in Indian buffaloes [13]. PPRV was also suspected to be involved in the epizootic disease that affected one-humped camels in Ethiopia in 1995–1996 [14] with detection of PPRV antigen and nucleic acid in some of the pathological samples, but no live virus was isolated. 

The genetics underlying this host-specific disease resistance to PPR is unknown. The two likely mechanisms are the differential presence or expression of viral specific receptors or the nature and type of the immune response. The signaling lymphocyte activation molecule (SLAM) a cellular receptor for PPRV, its expression level and PPRV replication rates have been shown to be highly correlated [15]. Furthermore, different levels of SLAM mRNA correlated with virus replication in different species such as cattle, buffalo, goat and sheep. In addition to SLAM, ovine nectin-4 was identified as a novel epithelial receptor for PPRV, which determines tissue distribution and pathogenicity [16]. The replication of PPRV in the PBMC of Indian domestic goats and water buffalo is influenced by the expression levels of TLR3, TLR7 and downstream signaling molecules. Upon stimulation of PBMC with synthetic TLR3 and TLR7 agonists or PPRV, the levels of pro-inflammatory cytokines were found to be significantly different across goats and water buffalo, a likely mechanism influencing differential susceptibility to disease [17]. In contrast, immunosuppressive interleukin (IL) 10 levels were lower in PPR-resistant Kanni and Salem Black breeds of goat and water buffalo at the transcriptional level, correlating with reduced viral loads in infected PBMC. In addition, water buffalo also produced higher levels of interferon alpha (IFNα) in comparison with goats both at transcriptional and translational levels which were confirmed to be TLR7 mediated through inhibitor and pre-treatment studies [17]. Thus, differential gene expression analysis can be a very powerful first attempt to correlate immune responses with gene regulation. Such approaches can also identify potential target genes for disease control. 

Earlier studies used candidate gene-based approaches (individual genes or proteins one at a time) to understand the host and pathogen interactions. To gain a more global understanding of gene expression underlying differential responses to PPRV infection, we used an RNAseq approach to study the transcriptome of goat and cattle PBMC exposed to PPRV in vitro. This systems biology approach may be useful in understanding differences in susceptibility toPPR in different animal species, identifying early markers of infection, potential antiviral targets and for understanding the basic molecular mechanisms of host-virus interactions.

## 2. Materials and Methods

### 2.1. Samples Used in the Study 

Blood samples for isolation of PBMC were collected from clinically healthy goats (Kanni cross, *n* = 6) and cattle (HF cross, *n* = 6) maintained at University Research Farm, Centre for Animal Production Studies, TANUVAS, Madhavaram Milk Colony, Chennai-51. These animals were not vaccinated for PPRV and sero-negativity was confirmed with an ID Screen PPR competition ELISA kit (ID.vet, Montpellier, France). The PPRV strain used for infection of PBMC was IND/TN/VM/2014/02 (at 10th passage level in Vero cells; GenBank Accession No. KT860063.1) for 24 h at different 1 MOI. RNAseq data was generated from two replicate PBMC samples from each individual in each species to act as biological replicates. Replicate samples from three animals were pooled together resulting in two samples from each species, control and PPRV infected.

### 2.2. RNA Isolation, Library Preparation and Sequencing

Twenty-four hours post infection was chosen as the most meaningful time point to study gene expression based on the replication kinetics data from our earlier studies with goats and buffalo PBMC [17] as well as those experiments with cattle and goat PBMC (Supplementary information—PPRV replication kinetics). RNAwas extracted from PBMC samples from each sample/species separately using Trizol as per the manufacturer’s instructions and cleaned up using RNeasy Mini kit (Qiagen, India) with DNAse I treatment. The RNA quality was assessed in Qubit^®^ 2.0 Fluorometer with the Qubit^®^ RNA BR Assay Kit (Life Technologies, Carlsbad CA, USA) and on a 2100 Bioanalyzer using an Agilent RNA 6000 Pico Kit (Agilent Technologies, Carlsbad CA, USA). For further processing, the RNA integrity number (RIN) values for all the samples were ascertained to be more than 9.0, the Ribo-Zero rRNA removal kit (Cat no. MRZH116, Illumina) was used to deplete ribosomal RNA with the protocol modified to suit the different RNA input amounts, the depleted RNA was purified with the RNA Clean and Concentrator–5 kit (Zymo Research, Irvine, CA, USA) to retain more than >17 nt RNA fragments and eluted in nuclease-free water. Libraries were constructed using depleted RNA obtained from total RNA using the TruSeq stranded mRNA sample preparation kit (Illumina, San Diego, CA, USA) with the depleted RNA introduced in the RNA fragmentation and priming step and the remaining steps performed as per the manufacturer’s protocols. The high-throughput paired-end sequencing was carried out on the Illumina HiSeq 2000 platform. The data generated from the two replicates (control and infected across both the species) were analyzed separately under the bioinformatic analysis projects P4365 and P4117 as provided in the results. 

### 2.3. Bioinformatic Analysis of the Sequence Data

#### 2.3.1. Quality Check and Assembly

A read quality check on the fastq file of the different samples was performed (which included base quality and sequence quality score distribution, read length and GC distribution, PCR amplification issues, any over-represented sequences and biasing of kmers) and sequences trimmed to retain high quality reads. Unwanted sequence including non-polyA tailed RNAs, mitochondrial genome sequence, transfer RNAs, adapter sequences and others were removed by using Cutadapt (V 1.8) and bowtie2 (version 2.2.2), in-house Perl scripts and picard tools (version 1.115). For the data generated from cattle PBMC (un-infected and PPRV infected) the *Bos taurus* reference genome and gene model downloaded from Ensembl database (ftp://ftp.ensembl.org/pub/release83/gtf/bos_taurus/Bos_taurus.UMD3.1.83.gtf.gz) and the pre-processed reads were then aligned using Tophat program (version 2.1.0; default parameters). After alignment with reference gene model, the aligned reads were used for estimating expression of the genes and transcripts, using cufflinks program (version 2.2.1). An in-house Perl script was used to generate a list of all detected splice junctions in the sample and the transcripts were scored differentially expressed at a *p* value of ≤0.01. Efficient and robust de novo reconstruction of the transcriptomes was also performed on the RNAseq data of goat PBMC (un-infected and PPRV infected) using Trinity (http://www.cs.huji.ac.il) with default settings. The trimmed reads were aligned to the assembled transcriptome using Bowtie2 programand the expression value as Fragments Per Kilo base of transcript per Million mapped reads (FPKM) distribution determined. For downstream annotation, we focusedonlyonthesetranscripts. The list of software, their version, source link and the purpose are listed in Supplementary Table (Table S1).

#### 2.3.2. Transcriptome Annotation, Differential Gene Expression Analysis and Protein-Protein Interactions

The assembled transcripts were compared with NCBI non-redundant protein database using BLASTX program. Matches with E-value ≤ 10^−5^ and similarity score ≥40% were retained for further annotation. The BLASTX summary is provided separately along with the e-value distribution. Around 58% of the transcripts identified using BLASTX have confidence level of at least 1 × 10^−5^, which indicates high protein conservation. The predicted proteins from BLASTX were annotated against NCBI, UniProt, Pathway and Swiss-Prot. The complete annotation is divided into three categories; gene, protein and gene ontology annotation. The aligned and annotated reads were also used for estimating expression of the genes and transcripts using cufflinks program (version 2.2.1) which provided data on gene and isoform expression in all the samples. The differential gene expression analysis was performed using the Cuffdiff program of cufflinks package and an FPKM > 1 for at least one of the two samples being compared with *p*-values of 0.01 and 0.05 separately were used as cut-off for up and down-regulated genes and isoforms respectively. The total number of transcripts mapping to some of the important sub-categories under the biological process (based on previous data) were selected for further analysis and validation to determine the differential expression across goats and cattle upon exposure to PPRV. Differentially expressed genes were submitted to the Search Tool for the Retrieval of Interacting Genes/Proteins (STRING) database (version 10.5) to identify the protein-protein interactions (PPI) and the network and spectrum of the gene interactome. 

#### 2.3.3. Validation of the Differential Targets Identified in RNAseq Data

Differentially expressed transcripts between goat and cattle identified in the RNAseq data were selected for validation by real-time PCR using SYBR Green chemistry. The selection of the transcripts was based on our previous report (differential interferon response as reported by Dhanasekaran et al., 2014) [17] as well as the PPI predicted by the STRING analysis. The real-time PCR (RT-qPCR) primers for the different targets were designed using the online tool Primer3 Input (version 4.0) targeting conserved regions across goat and cattle (Table 1). For this purpose, RNA was extracted from control and infected (1 MOI of PPRV) cattle and goat PBMC (*n* = 5) after 24 h to reinforce the significance and repeatability of the findings (refer Supplementary Information—*PPRV replication kinetics*) and cDNA was synthesized using the High-Capacity cDNA Reverse Transcription Kit with RNase Inhibitor (Thermo Fischer Scientific). Each sample was assayed in triplicate and expression levels were calculated as mean fold change 2^−∆∆Ct^ over the respective basal levels of each control sample. The RT-qPCR expression levels were considered significantly different if the *p* value was <0.05 and the results were also compared with the differential expression results predicted from the Cuffdiff analysis of the RNAseq data.

## 3. Results

### 3.1. Transcriptome Assembly, Annotation and Gene Ontology

#### 3.1.1. Transcriptome Assembly

**Figure 1:**
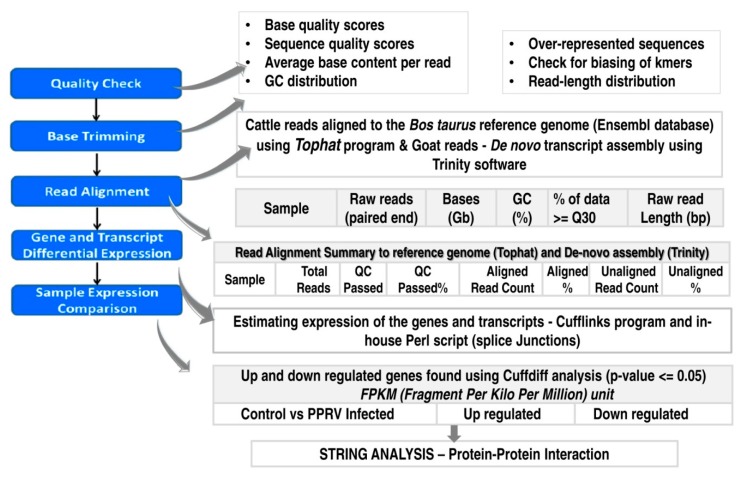
The bioinformatic workflow for analysis of the generated reads to determine the differentially expressed transcripts in peripheral blood mononuclear cells (PBMC) of goat and cattle upon infection with *Peste des petits ruminants virus* (PPRV).

The generated reads from the un-infected and PPRV infected goat and cattle PBMC were analyzed as per the bioinformatics workflow depicted in Figure 1. Raw reads were subjected to adapter and end trimming, filtering low quality data (Q < 20), discarding read length < 30 and contamination removal before assembly. The number of quality passed paired end reads was between 23.6 M to 30 M and 25.6 M to 25.9 M from each of the goat and cattle PBMC samples respectively(both un-infected and PPRV infected (Table 2). An average of 91.13% of the reads generated from each of the cattle and goat PBMC samplespassed at ≥30 Phred score (submitted to the Sequence Read Archive (SRA) database (BioProject ID-PRJNA615772)). The percentage of cattle reads aligning to the *Bos taurus* genome was 71.61% and 90.11% while de-novo assembly of the goat PBMC reads resulted in 86.83% and 87.77% alignment (meets the requirements as per the guidelines of ENCODE). The details pertaining to the quality passed reads, quality scores and the alignment count from the replicate (Bioinformatic analysis—P4117) are listed in Table 2.

#### 3.1.2. Annotation: 

With a minimum cut off value of ≥1 fragmentper kb of transcript per million mapped reads (FPKM) we could identify 352,462 transcripts with a read length of ≥200 bases (Table 3). The assembled transcripts were compared with the *Mammalia* protein database downloaded from Uniprot using the BLASTX program. The contigs without annotation were separated and compared with NCBI non-redundant protein database and matches with E-value ≤ 10^−5^ and similarity score ≥ 40% were retained for further annotation. We identified 50,063 (46.50%) assembled unique transcripts (longest identified transcript was 23,114 bases) withat least one significant hit in NCBI database (both BLASX and Uniprot) out of 352,462 predicted transcripts (length ≥ 200bp; FPKM ≥ 1.0) (Table 3). The BLASTX search E-value and the similarity score distribution is provided in Figure S1 and around 57%–58% of the transcripts identified using BLASTX had a confidence level of atleast 1e^-5^. We observed that 93% to 95% transcripts identified in BLASTX had a similarity of more than 60% at protein level indicating greater protein level conservation.

#### 3.1.3. Gene Ontology

Gene ontology (GO) analyses of the annotated transcripts were under taken as it indicates likely gene functions across cellular components, molecular functions and biological processes at a *p*-value ≤ 0.05. The total number of GO different terms identified across biological process, molecular function and cellular component included 33,412 (with 118,736 transcripts), 11,323 (with 71,161 transcripts) and 14,076 (with 81,646 transcripts) respectively. The top 15 terms in each of the above categories are depicted in Figure S1 (for detailed summary on the GO terms refer Supplementary Table; Table S2).

### 3.2. Functional Analysis of Differentially Expressed Genes across Goat and Cattle upon Exposure to PPRV

**Figure 2:**
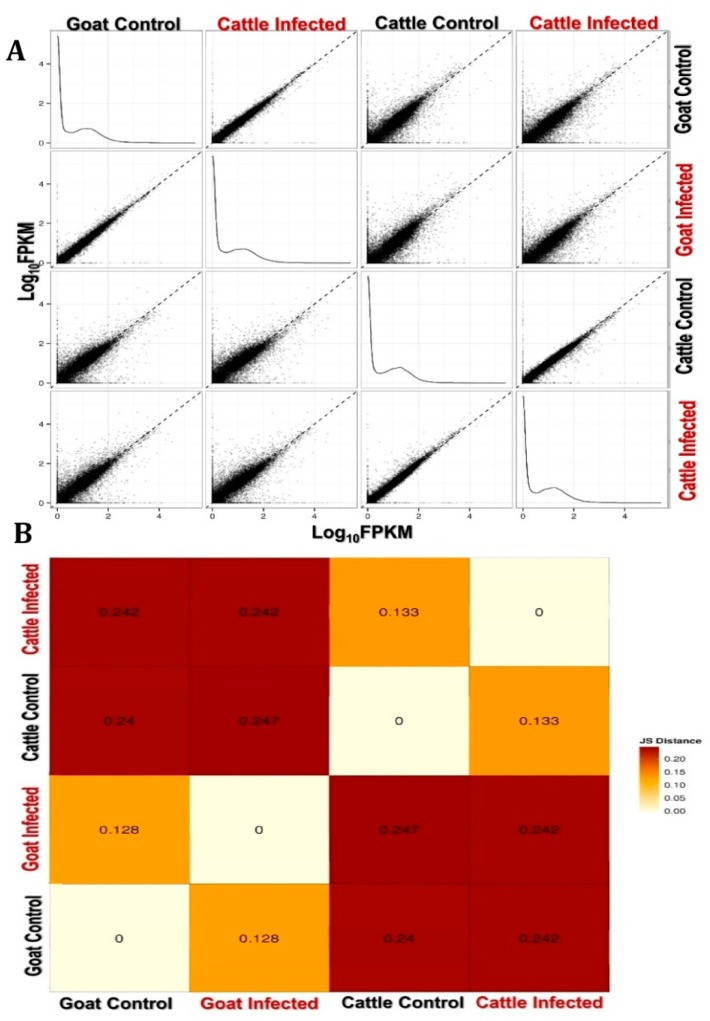
Correlation scatter and Distance Matrix plot of the estimated gene-expression across the samples compared in this study. (**A**) The estimates of the gene expression was performed using cufflinks and expressed as FPKM. The correlation scatter is depicted as Log_10_ FPKM across the samples. (**B**) The pairwise similarities across the samples are depicted as a matrix plot. Note: Both the scatter and the matrix plot clearly indicates the existence of a basal difference in the transcripts across goat and cattle PBMC as well as transcripts that are differentially regulated upon infection with PPRV.

One of the major applications of RNAseq experiments is a measure of the relative abundance of different transcripts (as FPKM) from the sequenced reads generated after transcriptome assembly. Differential gene expression analysis between goat and cattle before and after PPRV exposure was performed using the Cufflinks program. The gene-expression distribution in FPKM across the different samples is shown in Table 3, and confirms that even the low expressed transcripts (<100 FPKM) have been detected effectively in the data generated using both the reference genome (for cattle samples) and de-novo assembly (for goat samples) analysis. The longest transcript detected was 23,144 bases. Gene expression across all the samples are shown as a distribution plot, scatter plot and distance matrix plot that correspond to the pairwise similarities between the samples (Figure 2). The data clearly indicates differential transcript expression levels between species and upon exposure to PPRV. With default settings for the Cuffdiff analysis, the genes that are differentially expressed (up- and down-regulated) across Control vs. PPRV infected in the PBMC from goat and cattle PBMC at a *p* value ≤ 0.05 are shown in Table 4.

### 3.3. PPRV Infection Induces Different Immune Responses in Goats and Cattle

**Figure 3:**
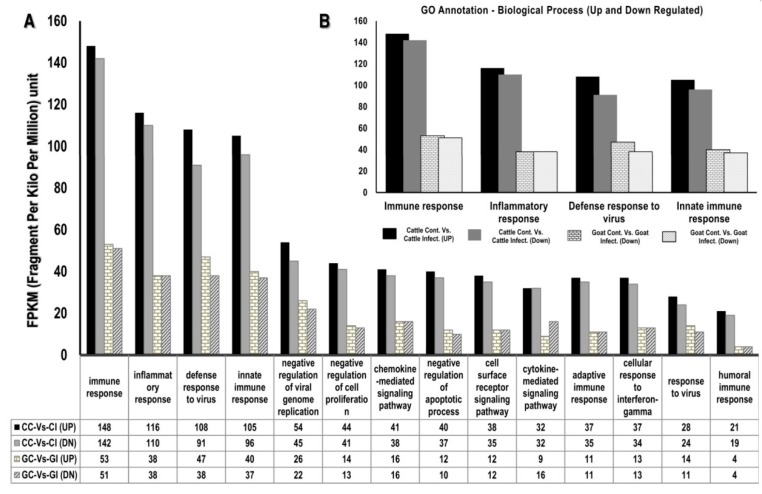
Gene ontology of the differentially expressed transcripts upon PPRV infection in goat and cattle PBMC. The gene ontology (GO) analysis was carried out for all the differentially expressed transcripts against all biological processes with a *p*-value ≤ 0.05. (**A**,**B**) The figure depicts the transcripts in FPKM for some of the selected sub-categories under the biological process (based on our previous report [17]) targeted in the study for further analysis to determine differential expression across goats and cattle upon exposure to PPRV.

**Figure 4:**
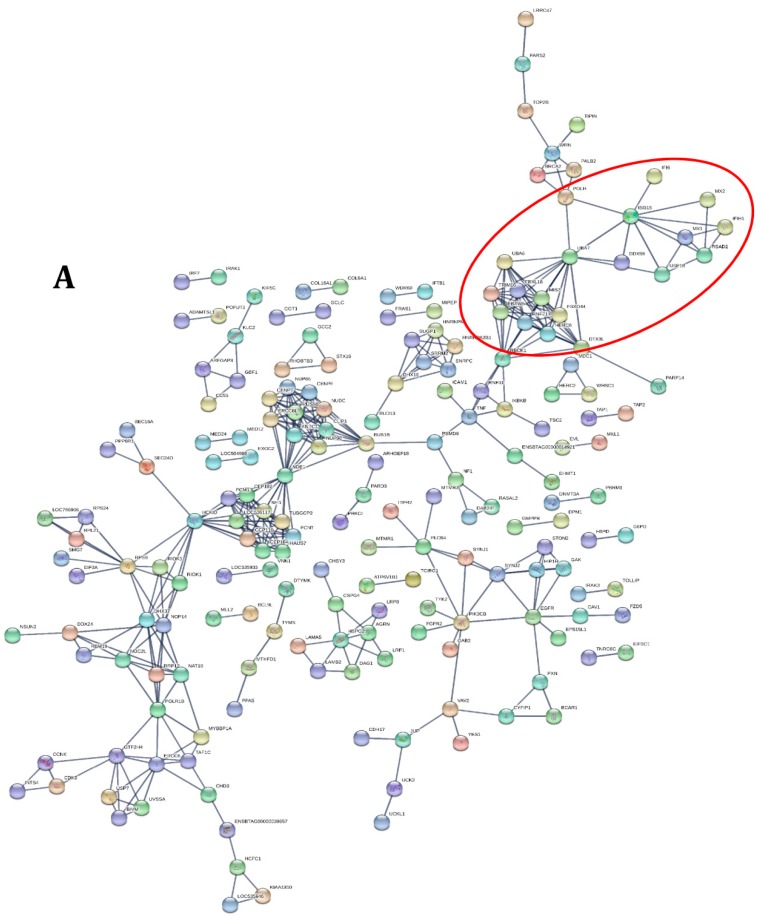

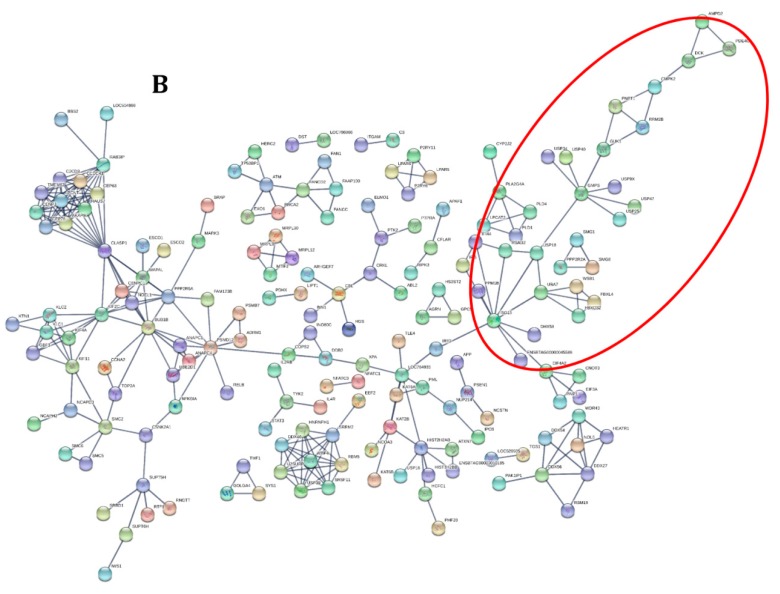
Protein–protein interaction (PPI) network for PPRV induced differentially expressed transcripts across goat and cattle by Search Tool for the Retrieval of Interacting Genes/Proteins (STRING) analysis. (**A**) Differentially expressed data of PPI from cattle PBMC; (**B**) Differentially expressed data of PPI from goat PBMC. Both the networks revealed the role of the interferon stimulated genes (ISGs) however, the network of proteins to be interacting were completely different. Those genes that play a role in the immune response, innate immune response, inflammatory response and defense response to virus are to be modulated by interferon stimulation. The different colors of the nodes are based on the scores for the co-expression, experimentally determined interaction, database annotation, text mining and the combined score. For reference on the scores the details are provided in the Supplementary Table (Table S5).

The GO analysis indicated that a great number of transcripts were up and down-regulated in cattle compared to goat and selected GO terms of genes contributing to immune response, inflammatory response and defense response to viruses are depicted in Figure 3A,B. STRING analysis of the differential expression data revealed eight different closely associated protein–protein interaction (PPIs) networks. As expected, the network of genes identified to be interacting was different between cattle and goat PBMC after PPRV infection (Figure 4A,B).

Across goat-control and goat-infected samples we identified 12 genes that were up-regulated and play a role in host defense against viruses with only one gene (macrophage mannose receptor (MMR)), responsible for binding and transmission of virus by macrophages, down-regulated (Table 5). The differentially regulated genes includedthe type I interferon stimulated effector genes namely interferon-induced 17-KDa/15KDa (ISG 17/ISG 15), Interferon-Induced Protein 44 (IFI 44), 2′-5′-Oligoadenylate Synthetase 1 (OAS1X), Interferon Alpha Inducible 6 (IFI6), Interferon-Induced Protein with Tetratricopeptide Repeats 1 (IFIT1), IFIT2 and IFIT3,MyxovirusResistance 1 (Mx1) and Myxovirus Resistance 2 (Mx2); Radical S-Adenosyl Methionine Domain-Containing Protein 2 (RSAD2) also called Viperin (mediating antiviral responses); DEXH (Asp-Glu-X-His) Box Polypeptide 58 (DHX58) that leads to Retinoic Acid-Inducible Gene I (RIG-I); gene-mediated antiviral response and Phosphodiesterase 12 (PDE12) which is an 2′,5′-oligoadenylate degrading enzyme that regulates the 2′,5′-Oligoadenylate Synthetase (OAS) enzymes and RNase-L pathway (Table 5).

However, in cattle control vs cattle infected, there were 22 genes that were up-regulated upon PPRV infection which included the type II interferon and the interferon gamma (IFNG); type I interferon stimulated effector genes namely ISG15, IFI44, OAS1X, IFI6, IFIT, IFIT2, IFIT3, Mx1 and Mx2, DHX58, OAS1X,2′-5′-Oligoadenylate Synthetase-1 (OAS1Y), Interferon-Induced Protein 44 Like (IFI44L), Interferon-Induced Transmembrane Protein 3 (IFITM3), RSAD2/Viperin, Interferon-Stimulated Exonuclease Gene 20 (ISG20), Interferon-Induced Protein with Tetratricopeptide Repeats 5 (IFIT5), DExD/H-Box Helicase 58 (DDX58), InterferonRegulator Factor 7 (IRF7), Interferon Induced With Helicase C Domain 1 (IFIH1) and PDE12; dsRNA binding induced serine/threonine protein kinase the Eukaryotic Translation Initiation Factor 2 Alpha Kinase 2 (EIF2AK2); and pro-inflammatory cytokine activated HECT and RLD Domain Containing E3 Ubiquitin Protein Ligase 5 (HERC5) (Table 6). The details on the role of the selected differentially expressed genes and their function in host responses against viral infection from published reports are listed in the Supplementary Table (Table S3).

**Figure 5:**
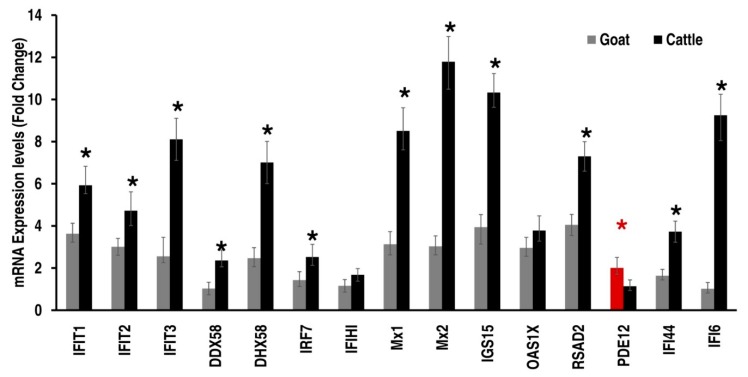
Validation of the differentially expressed ISG induced transcripts by RT-qPCR by SYBR Green chemistry. The PBMCs of goat and cattle were infected with PPRV at 1 MOI and RT-qPCR was performed on the 24 h PI, using the primers listed in Table 1. PPRV infection resulted in significantly increased levels of IFIT1, IFIT2, IFIT3, DDX58, DHX58, IRF7, MX1, MX2, IGS15, RSAD2, IFI44 and IFI6 in cattle, while PDE12 significantly increased in goat PBMC upon PPRV infection. (* indicates *p* < 0.05 determined by paired *t*-test and results of *n* = 5 samples).

**Figure 6:**
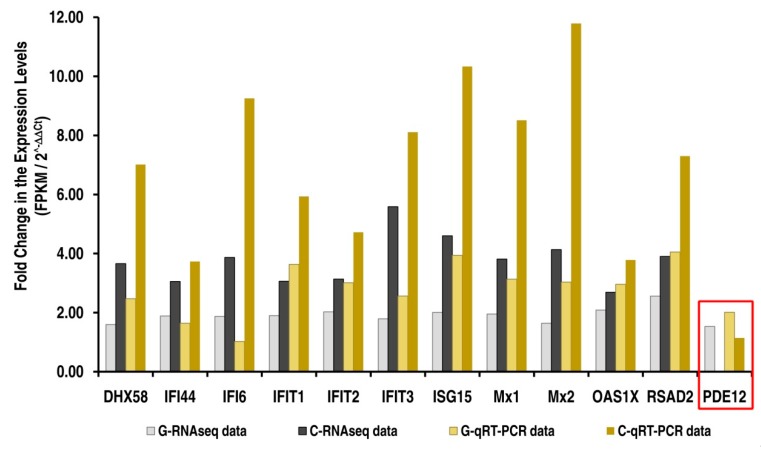
Comparing the expression levels selected differentially expressed target genes (RNA-Seq vs RT-qPCR) in response to PPRV infection in Goat vs. Cattle. The fold change in the FPKM values of the identified target genes from the RNAseq data has been compared with the fold change in the expression levels validated by RT-qPCR experiments. The expression levels of the negative regulator of the 2′,5′-oligoadenylate system probably contributing to the decreased antiviral state induced by the interferons is boxed in red; G, Goat; C, Cattle.

**Figure 7:**
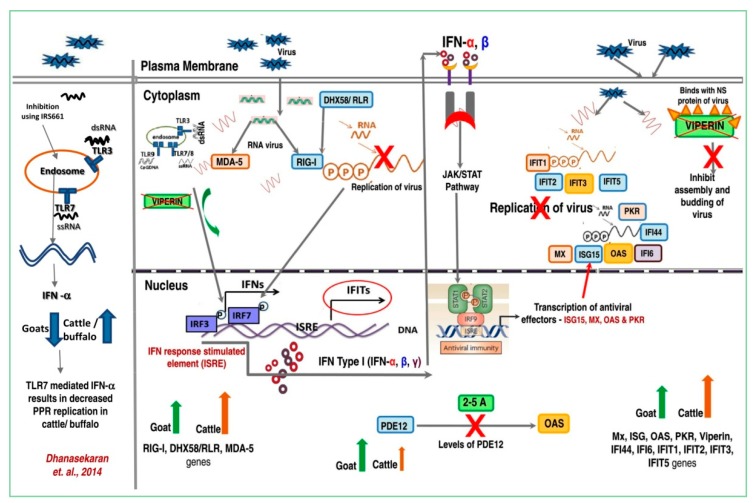
Postulated mechanism of disease resistance in cattle to PPRV—Schematic representation of roles of differentially expressed genes. The TLR7 mediated differential induction of RIG-I, DHX58, RLR and MDA5 contributes to the differential interferon levels which in turn modulate the viral replication across these two species. The increased levels of PDE12 might contribute to negative regulator of the OAS pathway in goats.

Among the 12 and 22 differentially expressed genes in goat and cattle that were identified from RNAseq data: In-vitro experiments were conducted to assess the expression levels of the IFI44, IFI6, IFIT1, IFIT2, IFIT3, ISG15, Mx1, Mx2, OAS1X, RSAD2, IRF7, DDX58, IFIH1, PDE12 and DHX58 by RT-qPCR. The interferon-induced genes, namely IFI44, IFI6, IFIT1, IFIT2, IFIT3, ISG15, Mx1, Mx2, OAS1X, RSAD2, IRF7, DDX58 and DHX58 were up-regulated in both species upon exposure to PPRV but with significantly higher levels in cattle which were also confirmed by RT-qPCR (Figure 5). However in goats, upon exposure to PPRV, the expression of Phosphodiesterase 12 (PDE12), a negative regulator of the 2′,5′-oligoadenylate system, is significantly increased, probably contributing to the decreased antiviral state induced by the interferons (IFNs) (Figure 5). The expression levels (FPKM) of the selected target genes identified by RNAseq correlated well with the RT-qPCR results, confirming the validity of the RNAseq data analysis (Figure 6; Supplementary Table, Table S4). We also have proposed a model depicting the roles of these differentially expressed target genes that contribute to the differential interferon levels which in turn regulates host responses resulting in differential PPRV replication in the goat and cattle (Figure 7).

## 4. Discussion

PPRV is capable of replicating in a wide variety of ruminant species but disease susceptibility is confined to small ruminants. Understanding the host factors that lead to viral pathogenesis and disease susceptibility or resistance will help to identify novel targets and inform better control strategies. In this study, we used RNAseq to study the increased susceptibility of goats to PPRV-induced disease compared to cattle by confirming increased viral replication and the identification and predicted function of differentially expressed genes induced during infection. Here we report the interaction of functionally annotated immune-related pathway genes that are differentially regulated between goat and cattle after exposure to PPRV.

In the past few years there has been a steady increase in the spread of PPRV across different countries and around 63% of the small ruminants globally may be under threat [18]. PPRV is considered small ruminant specific, with domesticated cattle and buffalo described as dead-end hosts. However, several surveillance programs have reported the presence of PPRV-specific antibodies in atypical hosts such as domesticated cattle (67%), buffalo (41%) and camel [11,19,20]. The potential of the virus to overcome innate resistance in these atypical hosts to result in evident clinical signs or disease transmission has been little studied [12,21]. The toll-like receptors (TLR) 7 and TLR3 play a major role in innate recognition of ssRNA viruses. Upon sensing the viral pathogen associated molecular patterns (PAMPS), a complex network of intra-cellular signaling pathways are activated by these TLRs which results in the production of several antiviral cytokines. Previous data from our laboratory clearly provides evidence that the viral recognition by these innate immune receptors and the subsequent cytokine profiles results in a 2log_10_ lower PPR virus titre in the PBMC of buffaloes when compared with goats [17]. Experiments on the replication kinetics of PPRV in cattle and goat PBMC revealed an increased virus titre and fold change in PPRV M gene RNA expression (0.2 log_10_ titre and 0.13 fold increase respectively) as early as 24 h post infection (PI) in goats (A 1.2 log_10_ higher virus titre and 3.81 fold increase in expression at 120 h PI in goat PBMC; for details refer to Supplementary Information—PPRV replication kinetics).

The genetic basis underlying differential host susceptibility or resistance is highly complex. Methods that enable the identification and analysis of gene interaction networksin a single experiment offer a powerful approach to understanding these complex processes. Systems biology approaches not only investigate the role of multiple genes contributing to a phenomenon but also generate information on their behavior and relationships in a quantitative, integrative and comprehensive manner [22]. High-throughput sequencing generated RNAseq data can provide gene expression profiles to identify genes likely to contribute to host-virus interactions and potentially to differential resistance/susceptibility across different species [23].

Among the cytokines, the interferons (IFNs) provide an anti-viral state to the host cells as well as help in modulating adaptive immune responses. Type I IFNs (IFNα/β) generate ananti-viral state by binding to the IFNα/β receptor which activates the transcription of interferon-stimulated genes (ISGs) via the JAK/STAT pathway while. Type II IFN (IFNγ) functions through a different receptor (IFNGR) to induce gamma activated factor (GAF) that activates the transcription of a distinct subset of cellular genes result in an antiviral state [24,25]. In addition to the interferon-induced genes, we also selected other significantly differentially regulated immune related genes with roles in the innate immune response, inflammatory response and defense response (Figure 3A,B) [17]. The classical inflammatory cytokines such as IFNβ, IFNγ, IL-4, IL-1β, IL-8, IL-10, IL-6 and IL-12 are induced in goats upon PPRV infection [26,27] even though the PPRV V protein possess the ability to inhibit the IFN signaling [28,29,30]. In experiments with the Sungri/96 PPRV vaccine virus, IFN-α/β mRNA were not detected after the infection of PBMC [31] and evasion of IFN-induced antiviral effects by PPRV have been shown to occur through miRNA mediated regulation of IRF3 and IRF7 [32]. In contrast, studies with a virulent PPRV strain demonstrated that genes within the interferon and RIG-1 like receptor signaling pathway, IRF-7 and STAT-1 that regulate the expression of ISGs, were active in lymphocytes [33]. Our previous report also indicates the contribution of TLR7 mediated higher IFN alpha induction as a factor for the lower replication of PPRV in cattle PBMC [17].

Viral infection-induced interferons induce an exclusive and moderately overlapping set of interferon stimulated genes (ISG) and studies have identified 50 to 1000 ISGs (using microarrays) that are typical to some cells types with effector functions limited to the classical ISGs, namely protein kinase (PKR; also known as eukaryotic translational initiation factor 2-alphase kinase -EIF2AK2), Myxovirus Resistance Protein 1 (MX1), 2′-5′-oligoadenylate synthetase 1 (OAS1), Tripartite Motif Containing 5 (TRIM5), Interferon-stimulated gene 15 (ISG15), adenosine deaminase acting on RNA (ADAR), interferon-induced transmembrane protein 1/2/3 (IFITM1/2/3 also called CD225), Tetherin (also known as BST2 or CD17), Viperin (also known as Radical S-Adenosyl Methionine Domain Containing 2 -RSAD2) [34,35] and ATP-dependent dsRNA helicase DHX58 also known as RIG-I-like receptor LGP2 (RLR) [36]. Up-regulation of the ISGs (ISG15, Mx1, Mx2, RSAD2, IFIT3 and IFIT5) that play a role in antiviral response and the viral sensors MDA5, LGP2 and RIG1 were found to up-regulated in lymphocytes upon exposure to a virulent strain of PPRV [33]. Our RNAseq data also reveal that the IFN type I responses are highly up-regulated in cattle. This increased type I interferon results in up-regulation of the ISG effector molecules such as Mx, OAS, PKR and related genes in cattle which have a crucial role to play in viral defense. One of the major effects of the IFN is induction of Oligoadenylate synthetase (OAS) that is capable of polymerizing ATP in a template-dependent fashion. OAS results in 2′,5′-linked oligoadenylate polymers (2-5A) which is not found in mRNA and DNA, resulting in the activation of RNase-L that leads to cleavage of viral genomes. This pathway is also regulated by enzymes encoded by virus and the host that degrades 2-5A. One such enzyme is phosphodiesterase 12 (PDE12) which degrades 2-5A and thus inhibition/decreased levels of PDE12 might up-regulate viral-infection-induced OAS/RNase-L pathway resulting in increased resistance to viral pathogens. Our results indicated that PDE12, a negative regulator of the innate immune response, is up-regulated in goats which could be a major reason for the decreased resistance to viral replication.

This host antiviral response is also potentiated by the increased levels of the interferon-induced IFIT family genes (IFIT1, IFIT2, IFIT3, IFIT5) in cattle (Table 6). The up-regulation of these genes was confirmed in the RT-qPCR experiments. In addition, it was observed that the DHX58/ RLR gene is also up-regulated in cattle. DHX58/ RLR induces RIG-I which helps in intra-cytoplasmic sensing of dsRNA and results in type I interferon release through the IRF-7 pathway. The stimulation of the IRF-7 gene has been shown toprovide host defense against viral infection by inducing Viperin/RSAD2 and our data shows that Viperin/RSAD2 are highly up-regulated in cattle. Gene profiling in IFN-α/βR-deficient mice indicates that Viperin is induced by several different viruses, dsRNA and poly-dAdT [37,38]. This pathway inhibits replication of Flaviviruses (HCV), West Nile virus (WNV) and Dengue virus, herpesviruses (HCMV), a paramyxovirus (Sendai virus), a rhabdovirus (VSV) an Orthomyxovirus (Influenza A virus) and a retrovirus (HIV-1) [39,40,41,42,43].

The fundamental aim of our study was to better understand differential resistance/ susceptibility to PPRV between small and large ruminants. Earlier reports from our work indicated that PPRV induced increased interferon levels in cattle and buffalo and were likely to be a major factor causing decreased PPRV replication. This study reveals a complex interaction of ISGs and their effector molecules is likely a major factor leading to host defense and decreased PPRV replication in cattle, confirming our earlier report. In summary, these results indicate the potential role of ISGs and their interacting proteins in the differential replication of PPRV in PBMC between goats and cattle. If the host immune responses in small ruminants are sufficiently suppressed, especially the interferon-stimulated genes, this could in part explain clinical disease in particular breeds and species. Further in-vitro experiments are being planned on selected target genes identified using a siRNA/ CRISPR Cas9 system approach and over expression to understand their contribution to the differential replication of PPRV between these two species. In addition, efforts to generate small molecule inhibitors to some of the targets identified, e.g., PDE12, could help in improving the immune responses to PPRV in the susceptible species.

## Figures and Tables

**Table 1:** The primer sequences, their location and the expected product size used for determining the expression levels of the gene targets in this study.

Gene Target	Sequence (5′----->3′)	Nucleotide Position	Product Size
PPRV-M-F	AGTGATTGAGGATAACGAC	3608–3627	242 bp
PPRV-M-R	TTAGCGCTAAACACACTTCC	3850–3830	
β-actin F	GGCTGTGCTGTCCCTGTAC	156–174	156 bp
β-actin R	CCGGAGTCCATCACGATGC	214–194	
GAPDHF	GGCGTGAACCACGAGAAGTATAA	470–492	119 bp
GAPDHR	CCCTCCACGATGCCAAAGT	588–570	
DHX58F	GGGACATGCTGAAGAAGCTC	723–742	175 bp
DHX58R	TTGTACCGACGCAGATGAAG	897–878	
IFI44F	GTCCATGTGGTGTTGCTCAC	1157–1176	180 bp
IFI44R	CTCCAGTTCCCACTCTGAGG	1336–1317	
IFI6F	AACTCGT/GTGGCCTCCTCAC	524–542	168 bp
IFI6R	TTCGACTTGCTTGTGGACAG	691–672	
ISG15F	CAGTTCATCGCCCAGAAGAT	147–166	171 bp
ISG15R	GTCGTTCCTCACCAGGATGT	317–298	
IFIL1F	ACTACGGCCGATTTCTGGAA	1467–1485	244 bp
IFIL1R	AGGGCCCGCTCATAATACTC	1709–1690	
IFIL2F	TGCTGCTATAGGGCCAAAGT	1084–1103	240 bp
IFIL2R	CACTTGTTTGGCTACG/AGGAG	1323–1304	
IFIL3F	GAGCTGGACTGTGAGGAAGG	461–480	242 bp
IFIL3R	AGGGCCAGGAGAACTTTGAT	702–683	
MX1F	GTCCCTGCTAACGTGGACAT	714–733	155 bp
MX1R	ACCAGGTTTCTCACCACGTC	868–849	
MX2F	GCAGATCAAGGCACTCATCA	992–1011	168 bp
MX2R	ACCAGGTCTGGTTTGGTCAG	1159–1140	
OAS1XF	CTGACCTCGTCGTCTTCCTC	260–279	224
OAS1XR	CAGGACATCGAACTCCACCT	483–464	
PDE12F	GTGTTTCGAATCAAGCAGCA	1186–1205	176 bp
PDE12R	CTCTGGAGCACCCTTTCTTG	1361–1342	
RSAD2F	ATTTGGACATTCTCGCCATC	570–589	182 bp
RSAD2 R	ATCCTCGTCCACGTTGAATC	751–732	
DDX58F	AAGGAT/CTGCCTCCATGACTG	2747–2766	164 bp
DDX58R	GCAGCATCAAATGGGATCTT	2910–2891	
IFI44LF	TTCCAAGGCCATTTGACTCG	798–817	150 bp
IFI44LR	CTCTTT/CCTCATCCAGCCCCA	947–928	
IFIH1F	ATCTCGTGTTTCAGGGCCAG	389–408	206 bp
IFIH1R	AGGGCCTCTACGAACATCCT	594–575	
IRF7F	TCCCCGCACTACACCATCTA	1614–1633	224 bp
IRF7R	AAGTGCTCCAGGAAGTGCTC	1837–1818	

These primers were used in a SYBR green based RT-qPCR to determine the expression levels of the corresponding target mRNA in the control and infected cells.

**Table 2:** The quantity and quality of the data generated in the next generation sequencing experiments.

Sample/SRA BioSample ID/SRA ID	No. of Paired End Reads and No. of Bases (Gb)	Aligned Read Count and Percent Aligned	% Q > 30	Number of Transcripts with FPKM ≥ 1.0
**NGS data generated from the Project–P4365**				
**Goat_4-Control:** [BioSample ID–SAMN14478390 & SRA ID–SRR11440511]	23,662,652 (4.73 Gb)	14,740,174 (86.83%)	90.62	179,233 **
**Goat_3- PPRV Infected:** [BioSample ID–SAMN14469261 & SRA ID–SRR11434618]	30,098,206 (6.01 Gb)	18,537,737 (87.77%)	91.36	150,093 **
**Cattle_4- Control:** [BioSample ID–SAMN14480722 & SRA ID–SRR11442915]	25,656,128 (5.131 Gb)	44,579,027 (90.11%)	91.3	11503
**Cattle_3- PPRVInfected:** [BioSample ID–SAMN14480403 & SRA ID–SRR11442832]	25,995,419 (5.199 Gb)	36,088,556 (71.61%)	91.25	11633
**NGS data generated from the Project–P4117**				
**Goat_2-Control:** [BioSample ID–SAMN14492330 & SRA ID–SRR11431701]	18,315,355 (3.66 Gb)	13,017,046 (89.76%)	87.95	83,315 **
**Goat_1-PPRV Infected:** [BioSample ID–SAMN14466456 & SRA ID–SRR11431292]	27,803,932 (3.54 Gb)	17,955,944 (88.27%)	87.87	71,874 **
**Cattle_2-Control:** [BioSample ID–SAMN14480879 & SRA ID–SRR11443109]	24,457,636 (3.08 Gb)	19,605,498 (63.68%)	87.54	10,414
**Cattle_1-PPRV infected:** [BioSample ID–SAMN14478687 & SRA ID–SRR11440572]	25,055,747 (3.23 Gb)	14,281,792 (57.00%)	89.64	10,356

SRA—Sequence Read Archive; FPKM—Fragments Per Kilo base of transcript per Million mapped reads; Project P4365—next generation sequencing to generate RNAseq data from the PBMC of goat and cattle (control and PPRV infected (replicate-1)); Project P4117—next generation sequencing to generate RNAseq data from the PBMC of goat and cattle (control and PPRV infected (replicate-2)); The raw reads generated for this project has been submitted to the NCBI SRA database under the BioProject ID–PRJNA615772 (the details on the BioSample ID and SRA ID are listed below each of the sample); These reads were subjected to adapter and end trimming, filtering low quality data (Q < 20), discarding read length < 30 and contamination removal before assembly and further analysis. The percent of the aligned read count for the samples to the genome are well within the requirements suggested by ENCODE consortium; **—transcripts predicted by de-novo assembly (due to non-availability of a completely annotated goat genome) and hence the number of transcripts with FPKM ≥ 1.0 is higher in the goat reads annotated.

**Table 3:** Transcript expression and total number of transcripts identified in the next generation sequencing experiments across goat and cattle PBMC infected with PPRV.

FPKM	Number of Transcripts (P4365) *				Number of Transcripts (P4117) *			
	Goat_4 Cont. **	Goat_3 PPRV Inf. **	Cattle_4Cont.	Cattle_3 PRV Inf.	Goat_2 Cont. **	Goat_1 PPRV Inf. **	Cattle_2Cont.	Cattle_1PRV Inf.
1–2	89,644	73,363	959	1048	44,664	32,444	1128	1111
2–5	46,460	37,026	1753	1706	16,919	17,975	2238	2287
5–10	17,944	17,794	1933	1914	9644	9809	2267	2215
10–20	14,126	12,592	2338	2398	6500	6250	2160	2000
20–50	8425	6863	2723	2700	3775	3522	1668	1611
50–100	1665	1524	978	1013	992	1009	473	561
≥100	969	931	819	854	821	865	480	571
Total	179,233	150,093	11,503	11,633	83,315	71,874	10,414	10,356
3,52,462 assembled transcripts (longest transcript 23,144 bases)					1,75,959 assembled transcripts (longest transcript 30,976 bases)			

Project P4365—next generation sequencing to generate RNAseq data from the PBMC of goat and cattle (control and PPRV infected (technical replicate-1). The raw reads generated for this project has been submitted to the NCBI SRA database under the BioProject ID—PRJNA615772 (the details on the BioSample ID and SRA ID are listed in Table 2); * Read length ≥200 bases; **: transcripts predicted by de-novo assembly and number of transcripts with FPKM ≥ 1.0 is higher in the goat reads annotated.

**Table 4:** Differentially expressed transcripts identified from the RNAseq generated across the Control and PPRV-infected Goat and Cattle PBMC using the Cuffdiff program of the Cufflinks package (*p*-value < 0.05).

Deseq Combination	Down-Regulated Transcripts	Up-Regulated Transcripts
Goat Cont. vs. Goat Inf.	474 (19.0%)	2017 (81.0%)
Cattle Cont. vs. Cattle Inf.	368 (68.0%)	173 (32.0%)
Goat Cont. vs. Cattle Cont.	1574 (60.7%)	1018 (39.3%)

(Data represented from the bioinformatic analysis under project p4365).

**Table 5:** The selected differentially expressed transcripts playing a role in innate immune response and host defense to virus in the Control vs. PPRV infected (Goat PBMC).

S.No	Gene ID	Gene	Goat Control FPKM Value	Goat Infected FPKM Value	log2 (Fold Change)
1	ENSBTAG00000007554	IFI6	685.79	2507.99	1.87
2	ENSBTAG00000007881	IFIT1	66.63	247.95	1.90
3	ENSBTAG00000008471	MX2	75.59	235.75	1.64
4	ENSBTAG00000009768	IFIT3	116.90	403.79	1.79
5	ENSBTAG00000014707	ISG15	725.86	2915.81	2.01
6	ENSBTAG00000016061	RSAD2	74.83	439.60	2.55
7	ENSBTAG00000030913	Mx1	266.28	1029.88	1.95
8	ENSBTAG00000034349	IFI44	41.14	151.84	1.88
9	ENSBTAG00000034918	IFIT2	144.59	588.48	2.03
10	ENSBTAG00000037527	OAS1X	60.86	258.11	2.08
11	ENSBTAG00000046580	DHX58	74.18	224.04	1.59
12	ENSBTAG00000015563	PDE12	19.27	51.96	1.43

(Data represented from the bioinformatic analysis under project p4365).

**Table 6:** The selected differentially expressed transcripts playing a role in innate immune response and host defense to virus in the Control vs. PPRV infected cattle (Cattle PBMC).

S.No.	Gene ID	Gene	Cattle Control FPKM Value	Cattle Infected FPKM Value	log2 (Fold Change)
1	ENSBTAG00000003366	DDX58	25.853	165.535	2.67873
2	ENSBTAG00000007554	IFI6	85.1433	1240.16	3.86449
3	ENSBTAG00000007881	IFIT1	63.5862	530.603	3.06085
4	ENSBTAG00000008142	IFIH1	55.5351	152.708	1.45931
5	ENSBTAG00000008471	MX2	34.5429	605.862	4.13253
6	ENSBTAG00000009768	IFIT3	7.60867	365.035	5.58425
7	ENSBTAG00000012529	IFNG	11.1059	39.2096	1.81988
8	ENSBTAG00000014707	ISG15	159.192	3860.52	4.59996
9	ENSBTAG00000014762	ISG20	90.9695	323.425	1.82998
10	ENSBTAG00000016061	RSAD2	41.7415	623.244	3.90024
11	ENSBTAG00000017367	IFIT5	22.1536	185.959	3.06937
12	ENSBTAG00000020538	HERC5	41.6011	155.056	1.89810
13	ENSBTAG00000030913	Mx1	50.5075	708.813	3.81084
14	ENSBTAG00000030932	IFI44L	49.6875	259.9	2.38700
15	ENSBTAG00000034349	IFI44	35.618	295.417	3.05208
16	ENSBTAG00000034918	IFIT2	14.4535	126.911	3.13432
17	ENSBTAG00000037527	OAS1X	56.6369	364.978	2.68799
18	ENSBTAG00000039861	OAS1Y	42.7186	970.258	4.50543
19	ENSBTAG00000046580	DHX58	11.4804	144.685	3.65567
20	ENSBTAG00000047680	IRF7	98.2855	457.14	2.21759
21	ENSBTAG00000008703	EIF2AK2	24.6231	92.6854	1.91233
22	ENSBTAG00000019015	IFITM3	215.423	683.86	1.66653

(Data represented from the bioinformatic analysis under project p4365).

## Supplementary Materials

The following are available online at https://www.mdpi.com/1999-4915/12/4/463/s1. Supplementary information: PPRV replication kinetics in goat and cattle PBMC. Figure S1: Transcript annotation of the goat and cattle transcripts that revealed BlastX matches to NCBI database (both as e-value and similarity distribution as percentage). Functional annotation of the transcriptome with BlastX matches to different GO category - Biological process; Cellular component and Molecular function (The top 15 GO terms are depicted in each of the above category). Table S1: List of software’s used for bioinformatic analysis of the data generated in the study. Predicted proteins from BLASTX were annotated against NCBI, UniProt, Pathway and other databases. Table S2: Functional annotation of the transcriptome with BlastX matches to different GO category. The details on the Gene Ontology ID, GO terms and number of transcripts are listed. The total number of GO different terms identified across biological process, molecular function and cellular component included 33,412 (with 118,736 transcripts), 11,323 (with 71,161 transcripts) and 14,076 (with 81,646 transcripts) respectively. Table S3: Details on the functional role of the differentially expressed transcripts in host response against viral infection identified in the study. Table S4: Relative expression levels of the differentially expressed transcripts in response to viral infection (based on the RNAseq data) using SYBR Green chemistry in of goat and cattle PBMCs infected with PPRV at 1 MOI (The depicted Ct values are mean of five samples tested; ∆Ct = target gene Ct- endogenous Ct; ∆∆Ct = ∆Ct of infected- ∆Ct of control; Fold change = 2^−∆∆Ct^). Table S5: The details on the PPIs identified in the STRING analysis of the differentially expressed targets in goat and cattle upon PPRV infection. The list of STRING interactions are listed in goat and cattle on separate sheets. The details on the scores for the co-expression, experimentally determined interaction, database annotation, text mining and combined score are used for the different colors of the nodes in the Figure 4A,B.

## References

[1] Lefevre P.C., Diallo A. (1990). Peste des petits ruminants. Rev. Sci. Tech. Off. Int. Epiz..

[2] Shaila M.S., Purushothaman V., Bhavasar D., Venugopal K., Venkatesan R.A. (1989). Peste des petits ruminants of sheep in India. Vet. Rec..

[3] Kulkarni D.D., Bhikane A.U., Shaila M.S., Varalakshmi P., Apte M.P., Narladkar B.W. (1996). Peste des petits ruminants in goats in India. Vet. Rec..

[4] Nanda Y.P., Chatterjee A., Purohit A.K., Diallo A., Innui K., Sharma R.N., Libeau G., Thevasagayam J.A., Burning A., Kitching R.P. (1996). The isolation of Peste des petitsruminants virus from Northern India. Vet. Microbiol..

[5] Shaila M.S., Peter A.B., Varalakshmi P., Apte M.P., Rajendran M.P., Anbumani S.P. (1996). Peste des petits ruminants in Tamilnadu goats. Indian Vet. J..

[6] Saravanan P., Sen A., Balamurugan V., Rajak K.K., Bhanuprakash V., Palaniswami K.S., Nachimuthu K., Thangavelu A., Dhinakarraj G., Hegde R. (2010). Comparative efficacy of Peste des petits ruminants (PPR) vaccines. Biologicals.

[7] Couacy-Hymann E., Bodjo C., Danho T., Diallo G.L.A. (2007). Evaluation of the virulence of some strains of Peste-des-petits-ruminants virus (PPRV) in experimentally infected West African dwarf goats. Vet. J..

[8] Singh R.P., Saravanan B.P., Dhar P., Shah L.C., Bandyopadhyay S.K. (2004). Development of a monoclonal antibody based competitive-ELISA for detection and titration of antibodies to Peste des petites ruminants (PPR) virus. Vet. Microbiol..

[9] Delil F., Asfaw Y., Gebreegziabher B. (2012). Prevalence of antibodies to Peste des petits ruminants virus before and during outbreaks of the disease in Awash Fentale district; Afar; Ethiopia. Trop. Anim. Health Prod..

[10] Anderson J., McKay A. (1994). The detection of antibodies against Peste des petits ruminants virus in cattle; sheep and goats and the possible implications to rinderpest control programmes. Epidemiol. Infect..

[11] Khan H.A., Siddique M., Sajjad U.R., Abubakar M., Ashraf M. (2008). The detection of antibody against Peste des petits ruminants virus in sheep; goats; cattle and buffaloes. Trop. Anim. Health Prod..

[12] Mornet P., Orue J., Gillbert Y., Thiery G.S.M. (1956). La peste des petits Ruminants en Afrique occidentale française ses rapports avec la Peste Bovine. Rev. Elev. Med. Vet. Pays Trop..

[13] Govindarajan R., Koteeswaran A., Venugopalan A.T., Shyam G., Shaouna S., Shail M.S., Ramachandran S. (1997). Isolation of Pestes des petits ruminants virus from an outbreak in Indian buffalo (*Bubalus bubalis*). Vet. Rec..

[14] Roger F., Guebre Y.M., Libeau G., Diallo A., Yigezu L.M., Yilma T. (2001). Detection of antibodies of rinderpest and Peste des petits ruminants viruses (Paramyxoviridae; Morbillivirus) during a new epizootic disease in Ethiopian camels (*Camelusdromedarius*). Rev. Med. Vet..

[15] Pawar R.M., Raj G.D., Balachandran C. (2008). Relationship between the level of signaling lymphocyte activation molecule mRNA and replication of Peste des petits ruminants virus in peripheral blood mononuclear cells of host animals. ActaVirol..

[16] Birch J., Juleff N., Heaton M.P., Kalbfleisch T., Kijas J., Bailey D. (2013). Characterization of ovine nectin-4; a novel Peste des petits ruminants virus receptor. J. Virol..

[17] Dhanasekaran S., Biswas M., Ambothi R., Ramya V.R., Raj G.D., Tirumurugaan K.G., Raja A., Kataria R.S., Parida S., Subbiah E. (2014). Toll-Like Receptor Responses to Peste des petits ruminants Virus in Goats and Water Buffalo. PLoS ONE.

[18] Baron M.D., Diallo A., Lancelot R., Libeau G., Margaret Kielian K.M., Thomas C.M. (2016). Chapter One—Peste des Petits Ruminants Virus. Advances in Virus Research.

[19] Ozkul A., Akca Y., Alkan F., Barrett T., Karaoglu T., Daglap S.B., Anderson J., Yesilbag K., Cokcaliskan C., Genacy A. (2002). Prevalence; distribution and host range of peste des petitsruminants virus; Turkey. Emerg. Infect. Dis..

[20] Abraham G. (2005). Antibody seroprevalences against Peste des petitsruminants virus in camels; cattle; goats and sheep in Ethiopia. Prev. Vet. Med..

[21] Khalafalla A.I., Saeed I.K., Ali Y.H., Abdurrahman M.B., Kwiatek O., Libeau G., Obeida A.A., Abbas Z. (2010). An outbreak of peste des petits ruminants (PPR) in camels in the Sudan. Acta Trop..

[22] Sharon D., Chenb R., Snyderb M. (2010). Systems biology approaches to disease marker discovery. Dis. Markers.

[23] Morrison-Smith S., Boucher C., Bunt A., Ruiz J. Elucidating the role and use of bioinformatics software in life science research. Proceedings of the 2015 British HCI Conference.

[24] Platanias L.C. (2005). Mechanisms of type-I- and type-II-interferon-mediated signalling. Nat. Rev. Immunol..

[25] Randall R.E., Goodbourn S. (2008). Interferons and viruses, an interplay between induction; signalling; antiviral responses and virus counter measures. J. Gen. Virol..

[26] Atmaca H.T., Kul O. (2012). Examination of epithelial tissue cytokine response to natural Peste des petits ruminants virus (PPRV) infection in sheep and goats by immunohistochemistry. Histol. Histopathol..

[27] Baron J., Bin-Tarif A., Herbert R., Frost L., Taylor G., Baron M.D. (2014). Early changes in cytokine expression in Peste des petits ruminants disease. Vet. Res..

[28] Fontana J.M., Bankamp B., Bellini W.J., Rota P.A. (2008). Regulation of interferon signaling by the C and V proteins from attenuated and wild-type strains of measles virus. Virology.

[29] Caignard G., Bourai M., Jacob Y., Tangy F., Vidalain P.O. (2009). Inhibition of IFN-alpha/ beta signalling by two discrete peptides within measles virus V protein that specifically bind STAT1 and STAT2. Virology.

[30] Manjunath S., Kumar G.R., Mishra B.P., Mishra B., Sahoo A.P., Joshi C.G., Tiwari A.K., Rajak K.K., Janga S.C. (2015). Genomic analysis of host- Peste des petits ruminants vaccine viral transcriptome uncovers transcription factors modulating immune regulatory pathways. Vet. Res..

[31] Ma X., Yang X., Nian X., Zhang Z., Dou Y., Zhang X., Luo X., Su J., Zhu Q., Cai X. (2015). Identification of amino-acid residues in the V protein of peste des petits ruminants essential for interference and suppression of STAT-mediated interferon signaling. Virology.

[32] Qi X., Wang T., Xue Q., Li Z., Yang B., Wang J. (2018). MicroRNA expression profiling of goat peripheral blood mononuclear cells in response to Peste des petits ruminants virus infection. Vet. Res..

[33] Wani S.A., Sahu A.R., Khan R.I.N., Pandey A., Saxena S., Hosamani N., Malla W.A., Chaudhary D., Kanchan S., Sah V. (2019). Contrasting Gene Expression Profiles of Monocytes and Lymphocytes From Peste des petits ruminants Virus Infected Goats. Front. Immunol..

[34] De Veer M.J., Holko M., Frevel M., Walker E., Der S., Paranjape J.M., Silverman R.H., Williams B.R.G. (2001). Functional classification of interferon-stimulated genes identified using microarrays. J. Leukoc. Biol..

[35] Sarasin-Filipowicz M., Oakeley E.J., Duong F.H.T., Christen V., Terracciano L., Filipowicz W., Heim M.H. (2008). Interferon signaling and treatment outcome in chronic hepatitis C. Proc. Natl. Acad. Sci. USA.

[36] Cui S., Eisenächer K., Kirchhofer A., Brzózka K., Lammens A., Lammens K., Fujita T., Conzelmann K.-K., Krug A.B., Hopfner K.-P. (2008). The C-terminal regulatory domain is the RNA 5’-triphosphate sensor of RIG-I. Mol. Cell..

[37] Olofsson P.S., Jatta K., Wågsäter D., Gredmark S., Hedin U., Paulsson-Berne G., Söderberg-Nauclér C., Hansson G.K., Sirsjö A. (2005). The antiviral cytomegalovirus inducible gene 5/viperin is expressed in atherosclerosis and regulated by pro-inflammatory agents. Arterioscler. Thromb. Vasc. Biol..

[38] Severa M., Coccia E.M., Fitzgerald K.A. (2006). Toll-like receptor-dependent and -independent viperin gene expression and counter-regulation by PRDI-binding factor-1/BLIMP1. J. Biol. Chem..

[39] Jiang D., Guo H., Xu C., Chang J., Gu B., Wang L., Block T.M., Guo J.-T. (2008). Identification of Three Interferon-Inducible Cellular Enzymes That Inhibit the Replication of Hepatitis C Virus. J. Virol..

[40] Jiang D., Weidner J.M., Qing M., Pan X.B., Guo H., Xu C., Zhang X., Birk A., Chang J., Shi P.Y. (2010). Identification of five interferon-induced cellular proteins that inhibit West Nile virus and dengue virus infections. J. Virol..

[41] Rivieccio M.A., Suh H.S., Zhao Y., Zhao M.-L., Chin K.C., Lee S.C., Brosnan C.F. (2006). TLR3 ligation activates an antiviral response in human fetal astrocytes, a role for viperin/ cig5. J. Immunol..

[42] Stirnweiss A., Ksienzyk A., Klages K., Rand U., Grashoff M., Hauser H., Kröger A. (2010). IFN regulatory factor-1 bypasses IFN-mediated antiviral effects through viperin gene induction. J. Immunol..

[43] Wang X., Hinson E.R., Cresswell P. (2007). The interferon-inducible protein viperin inhibits influenza virus release by perturbing lipid rafts. Cell Host Microbe.

